# A protocol for a pilot randomised controlled trial of an online cancer bereavement group

**DOI:** 10.1186/s40814-026-01785-y

**Published:** 2026-02-26

**Authors:** Lara King, Kirsten V. Smith, Charles L. Cole, Erin Hope Thompson

**Affiliations:** 1 Present Address: Division of Psychology & Lang Sciences, Faculty of Brain Sciences, Research department of Clinical Educational and Health Psychology (CEHP), University College London (UCL), London, UK; 2 Present Address: The Loss Foundation, [Registered Charity 1147362], London, UK; 3 Department of Experimental Psychology, University of Oxford, Oxford, UK; 4 Oxford Health NHS Foundation Trust, Oxford, UK

**Keywords:** Online bereavement groups, Therapist-led, Cancer loss

## Abstract

**Background:**

The loss of a loved one to cancer brings unique difficulties that affect the bereavement experience. Mixed evidence exists for the effectiveness of in-person group interventions for bereaved caregivers, with barriers to accessing support including perceived stigmatisation and geographical constraints. Online bereavement interventions offer an accessible means of providing grief support at the individual and group level. Research supports the effectiveness of online groups for those bereaved by cancer although most existing studies have examined peer-led online groups only, while empirical evidence on therapist-led online groups is still limited. This paper describes the protocol for a pilot trial evaluating the feasibility, acceptability, and exploratory effects of delivering online therapy groups for those bereaved by cancer.

**Methods:**

A longitudinal pilot randomised controlled trial (RCT) design will be used. A total of 100 adults who have lost a loved one to cancer will be randomised to receive the intervention immediately or after a delay three months later. Participants will engage in an eight-session online therapeutic group over 12 weeks, integrating cognitive-behavioural therapy, compassion-focused therapy, and grief models. Primary outcomes are feasibility (recruitment, retention, adherence, and data completeness) and acceptability (session attendance and participant ratings of helpfulness). Secondary outcomes include changes in grief intensity, depression, anxiety, post-traumatic stress, self-compassion, and social disconnection, measured at baseline, post-intervention, and three months post-intervention. Some measures such as depression, anxiety, grief intensity, and self-compassion will also be collected after each session. Feasibility and acceptability metrics will be assessed using pre-defined thresholds, and exploratory between-group differences in intervention outcomes will be examined.

**Discussion:**

This pilot trial will evaluate whether a therapist-led online group intervention is feasible and acceptable for adults bereaved by cancer. It is hypothesised that participants will show improvements in psychological outcomes post-intervention compared to waitlist controls, with effects being maintained at follow up three months later. Our findings will help to inform the design of a future full-scale RCT to establish efficacy.

**Trial registration:**

Feasibility and Outcomes of Therapist-led Online Cancer Bereavement Groups (NCT07002424), ClinicalTrials.gov, registered 3 June 2025, retrospectively registered https://clinicaltrials.gov/study/NCT07002424.

**Supplementary Information:**

The online version contains supplementary material available at 10.1186/s40814-026-01785-y.

## Background and rationale

Cancer is among the leading causes of death worldwide, accounting for 10 million deaths each year [8]. The loss of a loved one to cancer brings unique difficulties, such as the stress and trauma associated with the initial diagnosis, prolonged exposure to physical decline and caregiving demands. With such challenges, individuals bereaved by cancer are at increased risk of developing psychological difficulties including prolonged grief disorder (PGD), anxiety, depression, and post-traumatic stress disorder (PTSD) [28, 42, 45]. Given the profound psychosocial impact, there is an urgent need to develop acceptable and effective therapeutic interventions for this population.

Various barriers exist to delivering in-person bereavement support, such as fear of stigmatisation, geographical limitations, and restricted service availability [2]. Online bereavement interventions offer an accessible, destigmatising, and cost-effective means of providing grief support. Research into the effectiveness of online bereavement groups suggests that peer-led online groups offering emotional support and connection are insufficient in improving grief-related psychological outcomes [38]. A recent shift in evidence-based research has emphasised targeting underlying cognitive-behavioural processes known to maintain mental health difficulties following loss such as, maladaptive cognitions, rumination, avoidance, and trauma memories [40, 41].

A rapid review by Finucane et al. [16] found that online bereavement interventions involving therapist-guided CBT are feasible, acceptable, and effective in reducing grief, depression, and stress. Finucane et al. identified three therapist-guided interventions targeting cognitive-behavioural processes for cancer bereavement, all delivered individually rather than in group formats [20, 21, 50]. Therapist-led online bereavement group interventions were included, but none of these were cancer specific [32, 56]. Several quality improvement projects have described the development and implementation of online bereavement group interventions for individuals bereaved by cancer, including young adults and bereaved spouses [29, 30]. However, only one published study to date has evaluated a protocolised, group-based online intervention for cancer bereavement, focused on bereaved parents [33]. Our study offers a unique contribution by presenting, to our knowledge, the first protocolised therapist-led online group intervention for adults bereaved by cancer (beyond bereaved parents) that explicitly targets cognitive-behavioural mechanisms predictive of PGD.

The Loss Foundation is a national charity providing free support to individuals bereaved by cancer in the United Kindgom. Previous research demonstrated that its group-based cognitive-behavioural therapy (CBT) programme significantly improved self-compassion and reduced grief intensity, post-traumatic stress, depression, and anxiety among participants bereaved for at least six months, with a quasi-experimental waitlist control group showing no change at three-month follow-up [19]. This study analysed 27 participants in the intervention group and 11 in the waitlist group comprising mostly white females, with mixed relationships to the deceased. A larger service evaluation by Smith et al. [43] further supported these findings, demonstrating that The Loss Foundation’s CBT-based group intervention led to increased self-compassion and significant reductions in PGD, PTSD, depression, and anxiety, through targeting cognitive-behavioural mechanisms predictive of PGD (*N* = 68 participants across five cohorts). Cognitive-behavioural mechanisms included loss-related memory integration, negative appraisals, and maladaptive coping, suggesting that these processes may drive therapeutic change. Together, these findings provide growing empirical support for therapist-guided CBT-based group interventions as an effective approach for those bereaved by cancer.

The present study aims to ascertain the feasibility, acceptability, and changes in self-report measures of an online adaptation of The Loss Foundation’s group-based programme for individuals bereaved by cancer. Given the sufficient empirical foundation and its established theoretical basis in CBT, this programme was selected as a feasible choice for online adaptation. This pilot study will evaluate a randomised controlled trial (RCT) design to explore suitability for a further full-scale RCT. The intervention and waitlist (WL) control groups will be compared at baseline, after each session, and three months post-intervention on the following primary outcomes: grief intensity, depression, anxiety, post-traumatic stress, self-compassion, and social disconnection. Groups will include mixed types of cancer-related loss (e.g. spousal loss, child loss) due to resource constraints. While groups defined by relationship type can offer benefits, such as participants sharing similar loss circumstances, research supports the viability of mixed-loss groups, which may provide valuable perspectives, peer modelling, and facilitate recruitment and retention [15, 49].

### Aims and objectives

1. Our primary aim is to evaluate the feasibility and acceptability of delivering online bereavement groups for cancer loss. Feasibility will include the ability to recruit, randomise, and retain participants, while acceptability will be assessed through session attendance, participant satisfaction, and monitoring of adverse outcomes.
2. Our secondary aim is to explore the preliminary effectiveness by examining whether the online bereavement group intervention leads to improvements in various outcomes, compared to the WL control.

Our hypotheses are informed by the results of Jerome et al. [19] and Smith et al. [43], and previous literature suggesting that online groups may be similarly effective [33, 56]. Regarding the secondary aim of exploring preliminary effectiveness of the study, we hypothesise that grief intensity and PTSD symptoms, depression, and anxiety will be reduced post-intervention and self-compassion will be increased, showing improvement from baseline compared to the WL control. Further, we hypothesise that at follow-up, improvement will be maintained for grief intensity, PTSD symptoms, and depression.

## Methods: participants, interventions, and outcomes

### Trial design and setting

This study will use a longitudinal pilot RCT design to evaluate the feasibility, acceptability, and preliminary effectiveness of an online therapeutic bereavement group for adults bereaved by cancer. Participants will be randomly allocated to one of two parallel conditions: non-waitlist (Non-WL) whereby participants will access the intervention immediately and waitlist control (WL) whereby participants will receive the same intervention after a three-month delay.

Four groups will run online (two Non-WL and two WL) to ensure clinically appropriate group sizes and maximise therapeutic benefits. The groups will be identical in content and delivered in two parallel series to accommodate the WL design, enabling sufficient data collection within the study timeframe. The Non-WL groups will run first, over a period of 12 weeks comprising a total of eight online sessions. The first four sessions will take place weekly and the remaining four sessions fortnightly, allowing participants time to consolidate learning and apply skills between sessions. The WL groups will begin 3 months later, following the same schedule. Please see Supplementary Material 2: Appendix D for the research protocol.

### Eligibility criteria

Inclusion criteria are (1) adults (aged 18 or over) who are bereaved by cancer, for example losing a partner, family member, or friend; (2) the time since death must have been more than six months, so as not to interfere with a natural recovery process [48]; (3) self-referral to The Loss Foundation or via a related organisation; (4) have the means to access the therapeutic group online; and (5) participants consent to attending the intervention with the risk of a delay (e.g. by being randomised to the WL group).

Participants will not be eligible if they are (1) adults at high risk to themselves or others in regard to suicide and/or self-harm, (2) significant substance or alcohol misuse which would interfere with participants’ ability to take part in the research, and (3) cannot be involved in other therapy at the same time of the intervention elsewhere. Suicidal risk will be assessed during the telephone screening. Individuals assessed as high risk of harm will be referred immediately to The Loss Foundation’s in-house clinical psychologist for risk assessment and signposting to suitable support services.

### Intervention

The intervention will be delivered remotely via The Loss Foundation’s secure Zoom platform, each session lasting two hours. The intervention was developed by the second and last authors originally as a six-session weekly group intervention and has been refined over more than 15 years of clinical implementation and service user feedback. It was subsequently expanded to seven sessions to allow for more discussion and now includes an eighth session integrating the Continuing Bonds Theory [22], which supports maintaining symbolic and emotional connections following the loss of a loved one.

The intervention applies an integrative, transdiagnostic approach that targets psychological processes that maintain difficulties following bereavement such as avoidance, rumination, negative appraisals, emotional dysregulation, and self-criticism [44]. This approach aligns with research highlighting the overlapping psychological processes underpinning grief-related distress [6, 13]. The intervention is grounded in several complementary theoretical models. The dual process model of coping with bereavement [47] provides the overarching framework, balancing oscillation between loss-oriented and restoration-oriented coping. The model also draws on Tonkin’s [53] model of grief, which conceptualises adaptation as ‘growing around’ grief rather than resolving it.

The programme incorporates cognitive-behavioural mechanisms maintaining post-traumatic stress and prolonged grief [7, 12, 41, 43], which highlight difficulties integrating the loss into autobiographical memory and maladaptive appraisals of the self and world. Elements of compassion-focused therapy (CFT) [17] are incorporated to address self-criticism and shame, as well as promote self-compassion, and self-soothing. Together, these frameworks inform three central therapeutic components of the intervention: psychoeducation about grief and common psychological reactions; cognitive-behavioural techniques targeting avoidance, rumination, and distressing thoughts or memories; and compassion-based practices to strengthen self-care, resilience, and acceptance.

#### Structure and content

Each session will follow a consistent structure: a review of the previous week’s learning and action plan, introduction of new psychoeducational content, guided reflection, and a mindfulness or compassion-based exercise. Following each session, the research team will check in with participants to reinforce home tasks and self-care. Each session will have specific aims with the following structure:

- Week 1: introductions to the group and each other
- Week 2: self-compassion
- Week 3: the experience of grief, sleep difficulties and self-care
- Week 4: anxiety, low mood, avoidance
- Week 5: working with unhelpful thoughts and rumination
- Week 6: developing resilience to distressing memories
- Week 7: values and carrying our loved ones forward
- Week 8: reflections and endings

Criteria for discontinuing allocated interventions for a given trial participant: Any adverse outcomes defined as ‘sustained deterioration that is directly caused by the psychological intervention’ will be monitored throughout [11]. This will be measured via subjective report outlining any adverse effects on the self or others, collected after each session.

#### Facilitation and fidelity

Each group will include approximately 20 to 25 participants. Sessions will be co-facilitated by two trained clinicians (clinical psychologists and cognitive behavioural therapists) who volunteer at The Loss Foundation and have prior experience in bereavement support and group facilitation. All facilitators will complete a half-day standardised training and receive ongoing clinical supervision from Dr Erin Hope Thompson.

To ensure intervention fidelity, facilitators will adhere to a manualised protocol specifying content, structure, and timing. Fidelity checklists will be completed after each session, and any deviations or adaptations will be documented. Attendance will be tracked using Zoom reports, and participants will complete brief reflective exercises to reinforce learning and monitor engagement. The only expected modifications to the intervention relate to enhancing accessibility, for example for participants who are visually impaired.

### Comparator

It is possible that people in the WL control group feel frustrated if they must wait for the intervention rather than start immediately. Due to limited resources, The Loss Foundation does not have the capacity to run all the groups simultaneously, which presents a great opportunity to do an RCT given there will naturally be a delay for some starting the intervention. Participants in the WL group who consent for the delay in intervention will be given an electronic version of an information sheet containing signposting to organisations and self-guided information on grief and common signs of wellbeing declining (Please see Supplementary Material 1: Appendix B).

### Outcomes

#### Outcome assessment

Quantitative outcomes will be self-reported at baseline (T0) after each session (T1–T8) up to intervention completion and post-intervention three months later (T9). Participants in the WL control group will complete the baseline measures twice, initially (concurrent with the Non-WL group) and again before starting their intervention, to evaluate any change during the waiting period. The schedule of enrolment, interventions, and assessments is detailed in Table 1 (SPIRIT schedule).

**Table 1:**
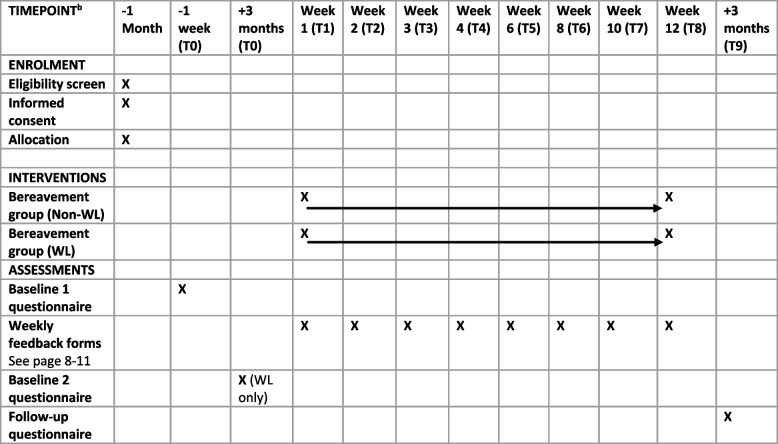
SPIRIT schedule of enrolment, interventions, and assessments [10]

Quantitative data will be collected via self-report measures assessing grief intensity, depression, anxiety, post-traumatic stress, self-compassion, and social disconnection. Data will be collected at baseline, post-intervention, and three months post-intervention, with selected brief measures administered weekly to track change across sessions. This repeated-measures design will allow exploration of when change might occur and which intervention components may contribute to improvement. All measures will be completed online through The Loss Foundation’s Qualtrics platform [36]. Participants will receive secure survey links by email, and automated reminders will be sent if questionnaires are incomplete. Completion of the longer pre- and post-intervention measures will take approximately 25 min, while weekly measures will take about six min. Participants will be informed of these time requirements and may complete surveys in more than one sitting if needed.

#### Primary outcomes: feasibility and acceptability

I. Recruitment and uptake
  Feasibility will be assessed through recruitment metrics, including referral source, number of individuals expressing interest, eligibility, consent, and randomisation rates. Consistent with guidance on feasibility studies, feasibility will be considered acceptable if ≥70% of the target recruitment is achieved within the planned recruitment period [14, 52].
II. Adherence and attrition
  Session adherence will be monitored via attendance records captured on Zoom and securely stored on The Loss Foundation server. A priori feasibility for session adherence is defined as ≥60%; feasibility of data completeness will also be assessed, with ≥70% of participants providing complete outcome data considered acceptable. Completion of outcome measures will be tracked using Qualtrics, with up to two reminder emails sent for missing post-session questionnaires.
  Strategies to promote retention will include emphasising the value of full attendance during screening, fostering a supportive group environment, encouraging sharing of reflections, providing brief between-session practice tasks, and sending follow-up reminder emails.
III. Acceptability
  Acceptability will be evaluated using the Helpful Aspects of Therapy (HAT) questionnaire [27], completed after each session to assess perceived helpful and unhelpful elements of the intervention. At the end of the programme, participants will also rate session length, online format, facilitator effectiveness, and group size using Likert-scale items [25]. Safety and adverse events will be monitored throughout.
  Pre-defined thresholds for acceptability are ≥70% of participants attending at least 50% of sessions and reporting positive ratings on session usefulness and format. HAT feedback will be summarised and shared with facilitators before each session to guide ongoing delivery and trial monitoring.
  Feasibility and acceptability outcomes will be compared against pre-specified traffic-light progression criteria ([4], see Supplementary Material 2: Appendix F).

#### Secondary outcomes: psychological outcomes

Aim: to assess the effectiveness of the therapeutic group intervention on cancer-bereaved adults’ grief intensity, depression, anxiety, PTSD, self-compassion, and social disconnection by comparing the Non-WL and WL control groups.

##### Pre- and post-intervention measures

The outcome measures used in this research are part of the standard data collection process undertaken by The Loss Foundation. The chosen measures for this research are informed by previous projects, which have shown evidence of positive change and are therefore being included to see if the online group has similar outcomes. This includes the measure of self-compassion as the therapeutic support group were based on CFT and aimed to enhance self-compassion.

The Patient Health Questionnaire-9 (PHQ-9; [23]) is a 9-item self-report questionnaire that measures the severity of depressive symptoms. Kroenke et al. [23] reported that the measure has strong psychometric properties: the internal consistency was 0.89 and test-retest reliability between patient self-report and mental health professional administering the measure on the phone 48 h later was 0.84. The measure also has good convergent validity (*r* = 0.73) with the mental health subscale of the Short-Form General Health Survey. The PHQ-9 has not been validated with bereaved populations.

The Generalized Anxiety Disorder-7 (GAD-7; [46]) is a 7-item self-report questionnaire that measures the severity of anxiety symptoms. Spitzer et al. [46] reported that the measure has strong psychometric properties: the internal consistency was 0.92 and test-retest reliability was 0.83 over a period of a week. The measure has strong convergent validity with the Beck Anxiety Inventory (*r* = 0.72) and the anxiety subscale of the Symptom Checklist-90 (*r* = 0.74). The GAD-7 has not been validated with bereaved populations.

Prolonged Grief Disorder-Revised (PG-13-R) [35] is a 13-item self-report measure assessing grief-related thoughts, feelings, and behaviours over the past month, including items on bereavement context, symptom frequency, and functional impairment. The measure has demonstrated strong psychometric properties; for example, Ashouri et al. [3] reported excellent internal consistency 0.93 and good test-retest reliability over a six-week interval (*r* = 0.89). The PG-13-R has also shown good reliability in identifying individuals at elevated risk for PGD, including in bereaved Swedish parents [34]. The PG-13-R was used as it aligns with DSM-5-TR diagnostic criteria for PGD, including the requirement that symptoms persist for at least 12 months post-loss, which was not captured in the original PG-13 [1].

The PTSD Checklist for DSM-5 (PCL-5; [57]) is a 20-item self-report questionnaire that assesses symptoms of PTSD. Blevins et al. [5] reported that the measure has strong psychometric properties: when tested with trauma-exposed undergraduate students, the internal consistency was 0.94 and the test-retest reliability was 0.82 over a period of a week. There appears to be no validation of the PCL-5 targeting traumatically bereaved individuals.

Oxford Grief-Social Disconnection Scale (OG-SD; [42, 45]) is a 15-item scale developed from interviews with bereaved individuals. Smith et al. [42, 45] indicate that internal consistency for the total OG-SD and its subscales was good or excellent (Cronbach alpha 0.80). The OG-SD showed good seven-day test-retest reliability across the total and subscales, indicating a temporally stable assessment of perceived social disconnection (test-retest 0.94). However, although overall the OG-SD was deemed stable over time, the other two subscales may be less stable over time.

The Self-Compassion Scale (SCS; [31]) is a 26-item self-report questionnaire that measures self-compassion. Raes et al. [37] reported that the measure has strong psychometric properties: the internal consistency was 0.85 and the test-retest reliability over a period of five months was 0.71. The total score of the SCS-SF correlates highly (*r* = 0.98) with the longer 26-item Self-Compassion Scale. Construct validity of the SCS-SF has not been tested, but the long version of the SCS was negatively correlated (*r* = −0.65) with the self-criticism subscale of the Depressive Experiences Questionnaire. The SCS-SF has not been validated with bereaved populations.

The Helpful Aspects of Therapy (HAT; [27]) is a post-session self-report questionnaire that asks about perceptions of key change processes in therapy. It is partly qualitative and captures perceptions of helpful and unhelpful aspects of the previous session. It is beyond the scope of this present study to analyse the qualitative data; however, this measure provides valuable feedback, which the facilitators can be aware of and address if appropriate.

#### Demographic data

As part of their group therapeutic interventions, The Loss Foundation routinely collects outcome measures and demographic information to evaluate the effectiveness of its services. For the therapy groups, the following data is routinely collected at varying time points across the intervention and securely stored: name; address (for participants requesting paper copies of the intervention manual); age; gender; ethnicity; marital status; highest level qualification; employment; psychological treatment since bereavement; gender of deceased. This data is considered ‘sensitive personal data’ under General Data Protection Regulation (GDPR); however, we believe there is a clear justification to report it as part of the evaluation, given that it will help us to better understand service access, including potential barriers to access.

Bereavement characteristics are also routinely collected by The Loss Foundation including: length of relationship with deceased (years, time since death); relationship with deceased; co-habiting with deceased prior to death; contact with deceased prior to death; frequency of seeing deceased in the three months preceding death; present at the death, death of other loved ones in past three years.

### Sample size

A total of 100 adults bereaved by cancer (50 per arm) will be recruited. As a pilot RCT, the sample size was selected to estimate key feasibility parameters (recruitment, consent, randomisation, adherence, retention, acceptability, and data completeness) with acceptable precision, in line with current guidance on pilot trial sample size determination, rather than to evaluate clinical effectiveness [14, 51, 54]. Although not powered for formal efficacy testing, the sample permits exploration of medium-to-large between-group differences, with any observed outcomes interpreted cautiously to inform outcome selection, variance estimates, and sample size planning for a future definitive RCT.

While online therapeutic groups are often smaller, anticipated attrition and high demand support initial recruitment of 25 participants per group. This size allows structured facilitation, participant engagement, and safety, consistent with prior evidence that slightly larger groups remain effective when appropriately organised [43]. Sessions integrate guided sharing, individual reflection, feedback monitoring, and psychoeducational content, making this format feasible while generating meaningful feasibility data to guide a future definitive RCT.

#### Non-adherence

To mitigate attrition, participants will be contacted regularly throughout the study and informed during consent that they may withdraw at any time. All participants will be screened via telephone to ensure their suitability and understanding of the study. In the event of withdrawal, participants will be followed up with to check on their wellbeing and provided with appropriate signposting support. While some non-response is expected in bereavement research, we will try to minimise missing data by sending reminder emails to prompt completion of follow-up measures.

### Recruitment

The Loss Foundation will send a promotional advert via their mailing list, website, and social media (Facebook and Instagram), inviting individuals to take part in the bereavement therapy groups. Please see Supplementary Material 2: Appendix A for a copy of the advertisement material. Basic information about the study and how to register interest will be displayed on their website and social media accounts. In addition, an email with a link to register interest in the study will be sent to all individuals on their mailing list. An email address for a member of the research team will be provided in these adverts. Participants will self-identify themselves by registering via a secure link and providing their preferred contact details. After self-referral, they will be invited to take part in a screening telephone call with a member of the research team to allow the team to fully explain the trial, to confirm eligibility, and to answer any questions. Following the screening phone call, individuals who give verbal consent for participation and who fulfil the inclusion criteria will be randomised in a 1:1 ratio to either the Non-WL or WL control groups. After randomisation and confirmation of their space in the programme, they will be invited to read the information sheet and sign the electronic consent form to give informed consent prior to participating (please see Supplementary Material 2: Appendix B). The information sheet given to participants includes details on randomisation to start the group immediately (Non-WL) or three months later (WL). Part of the eligibility criteria consists of participants giving informed consent to willingly be randomised including experiencing a delay in the intervention. Participants that do not consent to this will be excluded from the sample. Apart from the initial telephone screening, all correspondence with participants by the research team will be via email, unless the participant requests a telephone call or remote meeting to ask questions.

**Fig. 1:**
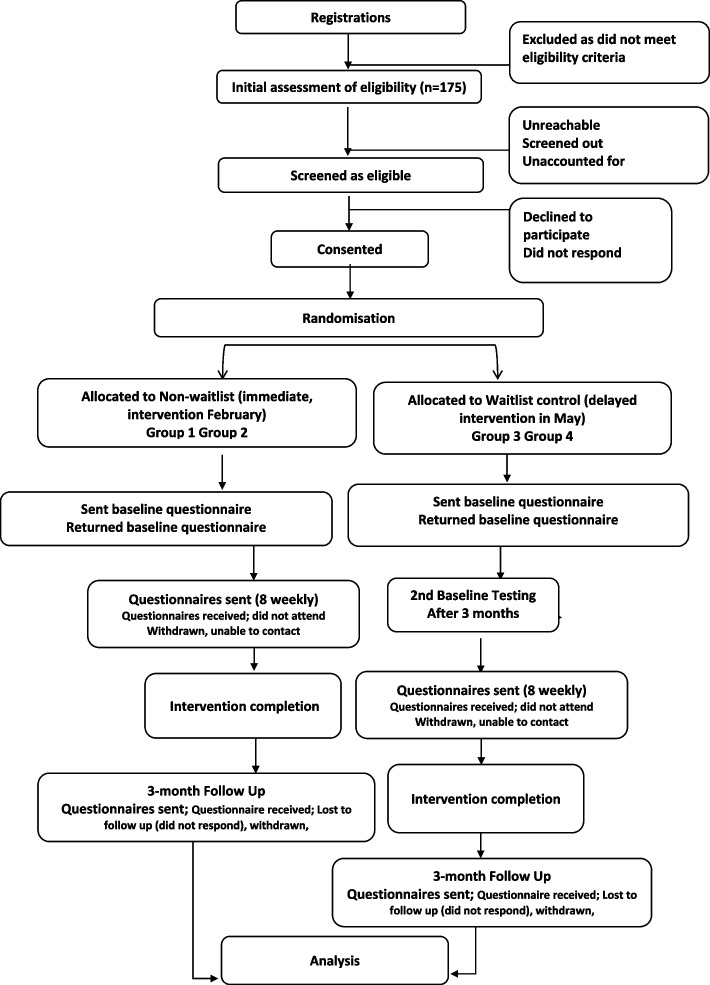
CONSORT flow diagram of participant timeline [14]

Participants will be recruited and assessed at baseline from February 2025 with data collection expected to finish in November 2025. Figure 1 shows the CONSORT flow diagram of the study participants.

## Methods: assignment of interventions

### Randomisation and allocation

Following telephone screening and verbal consent, eligible participants will be allocated to groups using a simple randomisation procedure via an online web-based system. Allocation will be communicated to participants by email. Due to the nature of the intervention, neither participants nor facilitators can be blinded to group assignment; however, participants will be asked not to disclose their allocation during outcome assessments. Following allocation, participants will receive an electronic information sheet and consent form, with all further correspondence conducted via email unless otherwise requested. We acknowledge how simple randomisation may result in unequal group sizes or imbalances in baseline characteristics, particularly in smaller samples; these will be examined descriptively and considered when interpreting exploratory outcomes [39].

## Methods: data collection, management, and analysis

All data will be stored on The Loss Foundation’s secure server. Survey administration will be automated through Qualtrics. Data will be pseudonymised and managed in accordance with the GDPR. Only the research team, including the principal investigator (Dr Charles Cole) and supervisor (Dr Erin Hope Thompson), will have access to anonymised data.

### Statistical analysis

All analyses will be conducted using JASP (2025, version 0.19.3) [18]. Data will be screened for outliers, missingness, and normality. Where assumptions for parametric models are moderately violated, we will proceed with them (as they are robust to mild non-normality), supplemented by non-parametric sensitivity analyses.

#### Primary analysis (feasibility outcomes)

The primary focus of this pilot trial is the evaluation of feasibility and acceptability outcomes, including recruitment and consent rates, retention and attrition, session attendance and adherence, and completeness of outcome data. These outcomes will be summarised descriptively using counts, percentages, and, where appropriate, confidence intervals. These data will be used to inform progression criteria and future trial design.

Progression criteria for a future definitive trial will be pre-specified using a traffic-light framework with green (go), amber (amend), and red (stop) thresholds. Thresholds were guided by best practice recommendations for external pilot trials [4] and informed by the expertise of the principal investigator and supervisor.

#### Exploratory secondary analysis (between-group)

Clinical outcomes (e.g. grief intensity, post-traumatic stress, depression, anxiety, self-compassion and social disconnection) will be analysed between the Non-WL and WL control groups only. The primary estimand is the difference in change between groups from baseline during the first three months. Linear mixed-effects models (or mixed ANOVA) will be used with fixed effects for group, time (baseline, three month follow up), and their interaction (group × time), and a random intercept for participants. The group × time interaction will index the treatment effect. Baseline scores may be included as covariates in an ANCOVA framework where appropriate. Effect sizes (standardised mean differences) and 95% confidence intervals will be reported.

If a sample size approaches *n* = 100, exploratory predictor analyses may examine associations between baseline characteristics (e.g. demographics, baseline severity) and outcome changes. This would be to inform outcome selection, covariate choice, and sample size calculations for a future trial. These analyses are descriptive and will not be used to draw confirmatory conclusions [24, 52].

#### Analysis population and missing data

Missing outcome data will be addressed using likelihood-based linear mixed-effects models, which use all available data and provide valid estimates under a missing-at-random (MAR) assumption [26, 55]. This approach is preferred over last-observation-carried-forward (LOCF), which relies on strong, often implausible assumptions and can bias estimates [9].

### Participant characteristics

Demographic and bereavement-related characteristics will be summarised descriptively for the overall sample and by group using means and standard deviations for continuous variables and counts and percentages for categorical variables. These data will be used to provide context for feasibility and acceptability outcomes, including service access and potential barriers. It may also inform exploratory analyses of potential covariates or predictors of change in clinical outcomes, while emphasising that such analyses are descriptive and hypothesis-generating only [14].

### Access to data

Only the study team (Dr Charles Cole- and members of The Loss Foundation directly involved in the research, including the primary supervisor (Dr Erin Hope Thompson) will have access to the data. The data processor will only have access to fully anonymised data. Any colleagues who have access to the data will be informed of the obligations to treat personal data confidentially and securely.

## Discussion

This pilot RCT primarily aims to evaluate the feasibility and acceptability of a therapist-led online bereavement group for adults who have lost a loved one to cancer. Feasibility and acceptability metrics, alongside pre-defined thresholds, will inform whether trial procedures and the intervention are suitable for a larger, definitive RCT. If the intervention is found to be feasible, acceptable, and preliminary data suggest potential benefits, a full-scale RCT could be justified. The group format may provide participants with opportunities to connect, share experiences, and potentially lead to improvements in psychological functioning across a range of bereavement outcomes. Findings from this study will also inform the refinement of the intervention, enhance trial procedures, and contribute to evidence-based practice for online bereavement support. More broadly, this research aims to advance understanding of effective cancer bereavement interventions and support the development of services that are responsive to participants’ needs and experiences. This aligns with UCL’s commitment to pioneering research informed by service user perspectives and to contribute to a growing evidence base.

Any amendments or updates to this protocol will be lodged with the journal such that it links them to this protocol document. This will allow all future trial publications and conclusions to be assessed against the extent to which we have adhered to the protocol.

## Supplementary Information

Supplementary Material 1.

## Data Availability

Data management and monitoring has been registered and approved by the Data Protection Office (reference No. Z6364106/2024/11/103 cancer research) in line with UCL’s Data Protection Policy. All anonymised data generated or analysed during this study are included in this published article (and its supplementary information files).
